# Appraising biocultural approaches to sustainability in the scientific literature in Spanish

**DOI:** 10.1007/s13280-023-01969-3

**Published:** 2024-01-24

**Authors:** Isabel Díaz-Reviriego, Jan Hanspach, Mario Torralba, Stefan Ortiz-Przychodzka, Camila Benavides Frias, Leonie Burke, María García-Martín, Elisa Oteros-Rozas

**Affiliations:** 1 Social-Ecological Systems Institute (SESI), Faculty of Sustainability, Leuphana University of Lüneburg, Universitätsallee 1, 21335 Lüneburg, Germany; 2 Environmental Geography Group, IVM Institute for Environmental Studies, VU University Amsterdam, Amsterdam, The Netherlands; 3 Land Change Science Research Unit, Swiss Federal Research Institute WSL, Birmensdorf, Switzerland; 4 University of Seville, Seville, Spain; 5 FRACTAL Collective, Madrid, Spain; 6 Social-Ecological Systems Institute (SESI), Faculty of Sustainability, Leuphana University of Lüneburg, Universitätsallee 1, C11.213, 21335 Lüneburg, Germany

**Keywords:** Biocultural diversity, Decolonization, Endogenous development, Epistemic justice, Indigenous and local knowledge, Latin America

## Abstract

Biocultural approaches that acknowledge the multiple and dynamic relationships between the diversity of cultures and nature are growing in popularity in sustainability research. Scientific contributions to biocultural approaches written in Spanish are numerous, including influential work on biocultural memory, biocultural heritage and biocultural ethics. However, despite linguistic diversity being considered essential in knowledge production for assuring broad and balanced evidence to successfully cope with sustainability challenges, non-English literature is rarely reviewed and taken into account in English-language scientific knowledge production and publications. This review assesses how the scientific literature in Spanish conceptualizes and applies biocultural approaches, showing their richness beyond the Anglophone predominance in academic knowledge production and communication. The results suggest that insights from Spanish-language scientific literature could contribute alternative methodological and theoretical pathways for biocultural approaches that might foster transformations for more sustainable human-nature relationships. We conclude by highlighting avenues that could bring more plural biocultural studies.

**Supplementary Information:**

The online version contains supplementary material available at 10.1007/s13280-023-01969-3.

## Resumen

Los enfoques bioculturales, que reconocen relaciones múltiples y dinámicas entre la diversidad de culturas y la naturaleza, son cada vez más populares en la investigación sobre la sostenibilidad. Las contribuciones científicas en español son numerosas, incluyendo trabajos sobre la memoria, la ética y el patrimonio biocultural. La diversidad lingüística se considera esencial en la producción de conocimientos para asegurar una base equilibrada de evidencias que permita afrontar los retos de la sostenibilidad. Sin embargo, rara vez se considera la literatura de habla no inglesa en la producción científica en inglés. Esta revisión evalúa cómo la literatura científica en español conceptualiza y aplica enfoques bioculturales, mostrando su riqueza más allá de la predominancia anglosajona en la producción de conocimiento académico. Los resultados sugieren que las perspectivas de la literatura en español podrían aportar propuestas teóricas y metodológicas alternativas. Concluimos destacando maneras que podrían contribuir a hacer más plurales las investigaciones bioculturales.

## Introduction

Biocultural approaches have received increasing attention in the academic literature over the last decades (Bridgewater and Rotherham 2019; Merçon et al. 2019; Lukawiecki et al. 2022). They emphasize the tight couplings of humans and their environments and champion the idea of biocultural diversity, which refers to the mutual adaptation between humans, non-humans, and their local environments. It encompasses the diversity of languages, cultural values, knowledge systems, customary stewardship practices, landscapes, and places that reflect the myriad ways in which people live with nature (Maffi 2005; Cocks 2010). A range of biocultural approaches to sustainability has emerged that acknowledges and studies the multiple relationships between the diversity of cultures and the maintenance, enhancement, and creation of biodiversity through practices and management of species, landscapes, and seascapes. Although the initial focus of most approaches was Indigenous peoples, local communities, and their lifeways, its use has been extended to urban areas and more dynamic understandings of culture (Cocks 2006; Mathez-Stiefel et al. 2007; Buizer et al. 2016; Elands et al. 2019; Vierikko et al. 2020; Stålhammar and Brink 2021). Biocultural approaches are seen as appropriate to address the current sustainability challenges across scales towards sustaining plural and just futures (Sterling et al. 2017; Pascual et al. 2021; Wyborn et al. 2021). Their applications are burgeoning in many scientific disciplines (Hanspach et al. 2020) as well as in civil society initiatives and international science-policy interfaces (Merçon et al. 2019; Hughes and Vadrot 2019; Hill et al. 2019).

Much work on biocultural approaches has been done in the Global South (Maffi and Woodley 2010). In Latin America, biocultural perspectives have inspired environmental and social justice movements as part of political struggles to protect Indigenous territorial rights and sovereignty over natural resources (Merçon et al. 2019). Latin American academics have also extensively contributed to the conceptualizations of biocultural diversity and the implementation of biocultural approaches, which has led to the publication of widely influential work on biocultural memory (Toledo and Barrera-Bassols 2008), biocultural heritage (Argumedo 2008; Boege 2008), and biocultural ethics (Rozzi 2012, 2013).

Our motivation for this review is to take into account linguistic diversity in knowledge production because we consider it essential for a more pluralistic account of the existent knowledge, and for a broad and balanced evidence-based to successfully cope with sustainability challenges (Coscieme et al. 2020; Lynch et al. 2021). Linguistic diversity is regarded as key for sustainability knowledge production since language is essential in meaning making and the way through which relationships between people and nature are interpreted and conceptualized. Therefore, linguistic diversity can expand the perspectives and understandings of those relationships and the range of options to tackle sustainability issues in context-appropriate ways (Droz et al. 2022, 2023), potentially also contributing to cognitive justice or the inclusion of different ways of knowing (Visvanathan 1997; Sousa Santos 2018).

However, language barriers and predominant patterns of knowledge production overlook linguistic diversity of the scientific evidence; internationally recognized scientific journals published in English rarely review and reference non-English scientific literature (Meneghini and Packer 2007; Tietze and Dick 2013; Amano et al. 2021a, b). While English as a lingua franca in contemporary scientific knowledge production facilitates communication and working together of scientists from different regions and cultures, (Woolston and Osório 2019; Lynch et al. 2021), it can also neglect other philosophies and ways of conducting research. In the case of biocultural approaches, recent reviews have looked at theoretical viewpoints, historical developments, discourses, and applications in the literature in English (Cocks 2006; Bridgewater and Rotherham; 2019, Hanspach et al. 2020, see Merçon et al. 2019 for an exception including some references in Spanish and Portuguese). To broaden and complement the perspectives of these previous assessments, we bring together and review biocultural approaches as reported in the scientific literature published in Spanish, which has a strong, though not exclusive, focus on knowledge produced in Latin America, a region of great importance to biocultural diversity worldwide (Loh and Harmon 2005). With this contribution, we aim to appreciate and take stock of the academic literature published in Spanish in order to make it more broadly accessible to non-Spanish speakers.

We assess how the scientific literature in Spanish conceptualizes and applies biocultural approaches. We do so by describing to what extent it engages with diverse types of knowledge, environmental values, and actors, among other things, and by creating different conceptual lenses that highlight different emphases that were taken. Based on the main insights from the scientific literature in Spanish, we discuss aspects in which these approaches converge and complement with those existing in the English-language literature as well as knowledge gaps. We conclude by highlighting possible avenues for future biocultural research. 

## Methods

**Fig. 1:**
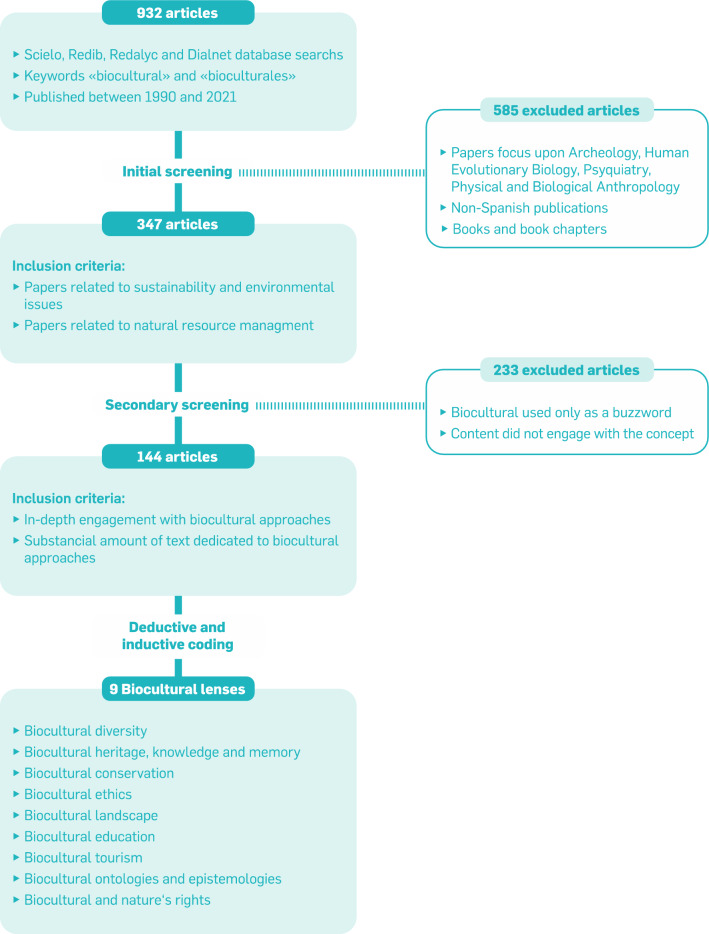
Flowchart describing the methodology for the selection of articles and for the identification of the 9 biocultural lenses

Our review utilized a systematic approach to bibliometric data collection and analysis. We searched Scielo (https://scielo.org), Redib (www.redib.org), Redalyc (www.redalyc.org), and Dialnet (https://dialnet.unirioja.es) databases for journal articles containing the Spanish keywords “biocultural” (singular) and “bioculturales” (plural) in October 2021. We selected these databases as they are the most relevant repositories of Spanish-language scientific literature. Only articles written in Spanish and published between 1990 and 2021 were included. The initial search yielded a total of 932 database entries. In a first round of screening, we checked title and abstract for relevance, the link to sustainability, and excluded all duplicates. For example, we excluded articles from unrelated disciplines such as Human Evolutionary Biology and Physical and Biological Anthropology. We assumed a link to sustainability when a publication jointly considered interlinked social and environmental issues. This first screening yielded a subset of 347 publications for which we reviewed the full text. In this second round of screening, we excluded all publications that used “biocultural” only as a buzzword. That means, we excluded publications that only mention “biocultural” in passing (often only in the keywords or the abstract) and that did not dedicate larger shares of the text to engaging with the concept. The second screening provided the final sample of 144 publications (Fig. 1). See Supplementary information for a list of the reviewed papers. We acknowledge that by limiting the search to only publications that explicitly mention the term “biocultural,” we exclude a wide range of studies that engage with relevant aspects. This includes, for example, the literature on Indigenous and local traditional ecological knowledge (see Brondízio et al. 2021 for a recent review). Also, focusing on publications in Spanish overlooks much academic work published in other languages. Nevertheless, we focus on shedding light on the dynamic and growing literature in Spanish, a lot of which is produced in Latin American countries with bioculturally diverse areas.

The review process was a combination of deductive and inductive coding. We first coded deductively for 10 predefined variables to broadly characterize the different publications. The variables are fully described in Table 1 and were taken from a recent review on biocultural approaches in the English literature (Hanspach et al. 2020). We calculated descriptive statistics for each of the variables. We then coded publications for different ways of understanding and applying biocultural approaches or “biocultural lenses.” For that, the lenses from Hanspach et al. (2020) formed the initial basis (Table 2), but they were adapted to represent the perspectives of the literature in Spanish. New lenses were created inductively when necessary. The coding of lenses was split between the co-authors and inconsistencies or doubts were iteratively discussed. A subsequent round of coding of the lenses was done in order to ensure consistent and reliable results. Since articles could represent aspects from different lenses, we distinguished between associating to a single primary lens, i.e., the main association of a paper to a lens, and secondary lenses, i.e., multiple other lenses that a paper could be associated to. We examined the lenses patterns over time in order to consider the growth and evolution of the conceptualizations of biocultural approaches. The description of a given lens in the results section was based on articles associated to that lens as primary lens. Subsequently, we quantitatively analyzed the primary and secondary lenses and publication characteristics using a multivariate analysis. Specifically, we applied a detrended correspondence analysis (DCA) of the lenses based on a matrix with a value of 1 for a primary lens and 0.5 for secondary lenses. The DCA arranges the publications in a multivariate space according to their similarity in the lenses that they are assigned to. Of this usually, the first two dimensions are interpreted (see Fig. 4). In order to understand how the resulting pattern relates to other characteristics of the papers we used a post hoc test to assess the correlation of the first two ordination axes with the quantitative characteristics (i.e., all variables from Table 1). For this, we applied a permutation test with 9999 permutations and a significance level of 0.05 using the *envfit* function from the *vegan* package (see Supplementary information for detailed results). All quantitative analyses were performed in R version 4.1.2.

**Table 1:** Variables used for deductive coding and descriptive statistics

Name	Description
Type of paper	What type of paper is it? (conceptual, discussion, empirical, review)
Emphasis	Does the study mainly focus on cultural (or social) aspects or on biological (or ecological) aspects? (purely cultural, mainly cultural, balanced, mainly biological, purely biological)
Focus	Does the study emphasize preservation/conservation or dynamic/transformation of biocultural components? (conservation, balanced/mixed, transformation)
Knowledge type	Which type of knowledge does the paper focus on? (local/traditional; mixed; scientific)
Value type	Which type of environmental value (Chan et al. 2016) does the paper focus on? (instrumental, relational, intrinsic)
Power	Does the study consider power relations? (yes/no)
Gender	Does the study consider gender dimensions? (yes/no)
Action	To which degree is the paper a call for participatory action? (not mentioned, mentioned but not the main focus, action is the main focus)
Governance	Which types of governance and decision making is emphasized? (not considered, bottom-up/decentralized, polycentric/multilevel, top-down/centralized)
Participation	To what extent are non-academic actors involved in the research process (Brandt et al. 2013)? (no involvement of non-academic actors, informed by/consultation of non-academic actors, collaboration with /empowerment of non-academic actors

**Table 2:** Biocultural lenses from Hanspach et al. (2020) that were used as the basis for coding

Biocultural lens	Short description
Biocultural diversity	Conceptualizes or describes the tight coupling between humans and their environment through the concept of biocultural diversity
Biocultural conservation	Focuses on the conservation of biocultural diversity or of nature with biocultural methods
Biocultural landscape and natural resource management	Emphasizes a spatial perspective of the tight coupling of humans and their environments
Biocultural history and heritage	Highlights the temporal dimension and outcomes of the long history of human–environment interactions
Biocultural knowledge and memory	Focuses on knowledge, practices, beliefs and values as expressions of biocultural diversity
Biocultural ethics, rights and sovereignty	Emphasizes issues around justice, rights and sovereignty of local or Indigenous people
Biocultural restoration, transformation and design	Focuses on the use of biocultural approaches to guide and implement change towards desirable futures

The applied methodology of coding for different lenses gives a broad classification of how a given biocultural approach is applied. This is arguably only one out of many possible ways to answer our research question and can only be a first step to exploring the richness of the biocultural literature in Spanish. As described above, our study builds on a previous review by Hanspach et al. (2020), which shares some of the authors of this paper. Between the two, some aspects are similar, some are further developed and some are different. For example, there is a great similarity in the search string, the selection procedure for the articles, the coding of the descriptive variables, and the quantitative data analysis. It is different by further developing the biocultural lenses presented by Hanspach et al. (2020) and by adapting these to the themes in the literature in Spanish, and by the fact that the literature search was done in different databases that are more suitable for finding articles written in Spanish.

## Results

### General overview

**Fig. 2:**
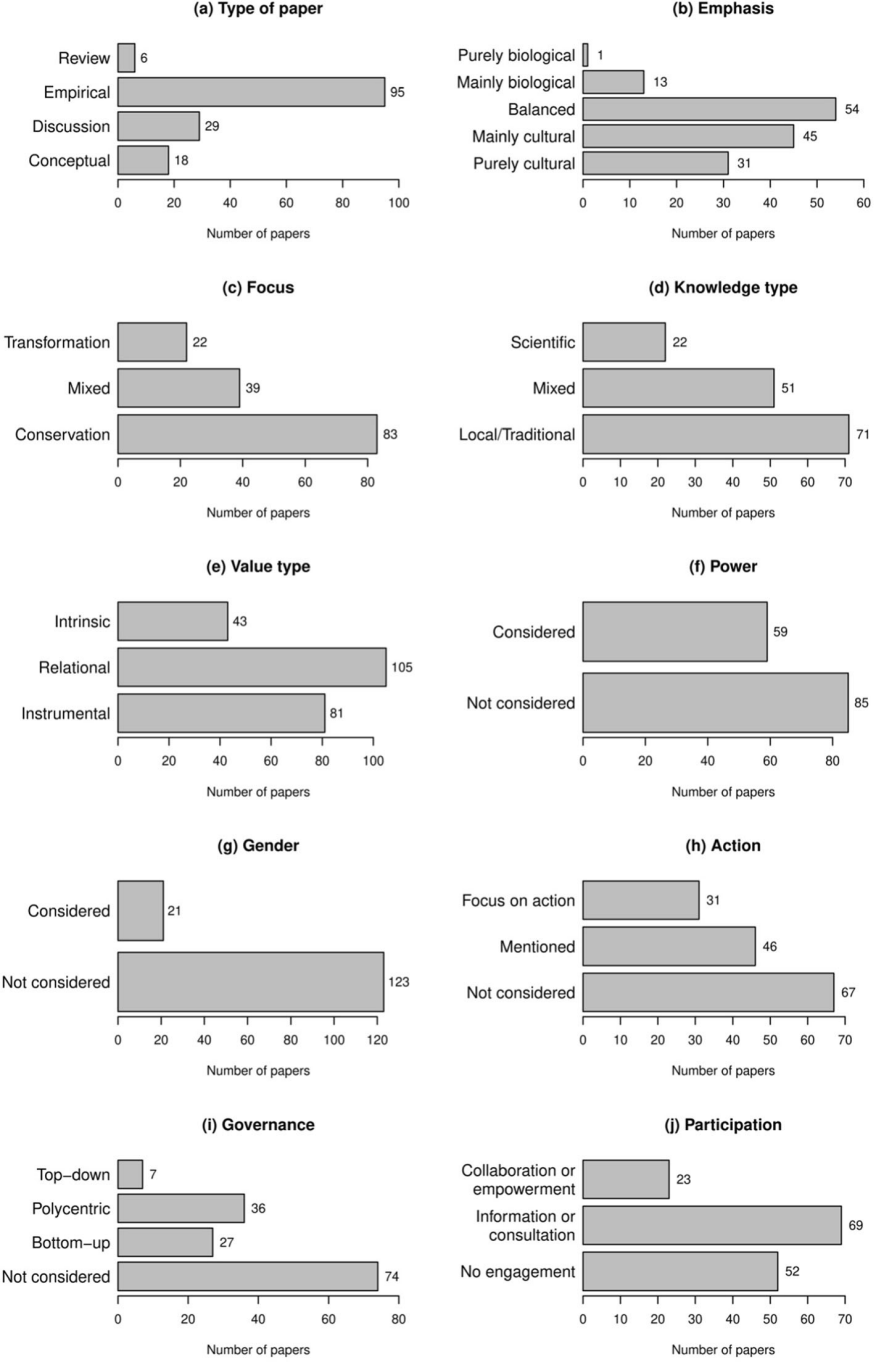
Descriptive overview of the characteristics derived through deductive coding. This includes **a** type of paper, **b** main emphasis, **c** focus, **d** knowledge type, **e** environmental value type, **f** consideration of power, **g** consideration of gender, **h** consideration of participatory action, **i** governance type, and **j** degree of participation

We reviewed 144 scientific publications in Spanish with a main focus on biocultural approaches to sustainability. Two thirds of the publications were empirical (*N* = 95, Fig. 2a), and we observed an emphasis towards considering either biological or cultural aspects (*N* = 54; Fig. 2b) or mainly cultural aspects (*N* = 45; Fig. 2b). Biocultural conservation was the focus taken by the majority of publications (*N* = 83; Fig. 2c). Local and traditional knowledge types (*N* = 71; Fig. 2d) and relational values through which people give more importance to meaningful relationships with nature (Chan et al. 2016; Stålhammar and Brink 2021) (*N* = 105; Fig. 2e) were most often considered. While power issues were studied in more than half of the publications (*N* = 85, Fig. 2f), gender issues were neglected to a great extent since these were only assessed in very few publications (*N* = 21; Fig. 2g). Participatory action was mentioned in about a third of the sample (*N* = 46; Fig. 2h); however only, a limited number of the articles analyzed (*N* = 31; Fig. 2h) had participatory action as focus. Governance was not considered in about half of the papers reviewed (*N* = 73, Fig. 2h), and the papers that considered it was most often as polycentric (*N* = 36; Fig. 2i) and bottom-up (*N* = 28; Fig. 2i) governance. The participation of non-academic actors in the research process often happened through information and consultation (*N* = 69; Fig. 2j).

### Biocultural lenses

The assessment of the articles led to the identification of 9 lenses based on the distinctive emphases of the biocultural approaches in the literature in Spanish. Each of these perspectives represents a particular vantage point, offering a different conceptual ‘lens’ to make sense of the complex and dynamic relationships between biological diversity and cultural diversity. Indeed, these lenses are generally overlapping in the articles analyzed. This delineation is based on our own qualitative interpretation of the literature reviewed.

#### Biocultural diversity lens

This lens entails articles that conceptualize and describe biocultural diversity. Different aspects are considered, including the ways in which humans and their environments are intertwined (Luque Agraz and Doode Matsumoto 2009; Ojeda et al. 2018), the interactions between cultural diversity and biological diversity (Ramos Roca and Corona-Martinez 2017) and the spatial overlap between these diversities (Tetreault and Lucio López 2011). Articles within this lens usually include an empirical place-based component. The empirical parts usually examine biocultural diversity in (a) certain contexts, for example, in relation to different land uses such as agroforestry (Moreno-Calles et al. 2013) and homegardens (Calvet Mir et al. 2014; Martínez et al. 2020; Castello et al. 2021); (b) species groups like cactaceae in Cárdenas Camargo (2019), cassava in Pérez et al. (2019), and native stingless bees in Vásquez-García et al. (2021) and Chan Mutul et al. (2019); and (c) management practices of specific social groups like Comcáac Indigenous people in Mexico in Luque Agraz and Doode Matsumoto (2009), the Shuar in Ecuador in Paño Yáñez (2020), and transhumant cattle farmers in Argentina in Califano (2020a, b). Particularly, food markets are considered as an important place for the study and exchange of biocultural diversity (Cababié et al. 2015; Argueta Villamar 2016; Colin-Bahena et al.^2018^; Puentes et al. 2020). This lens also acknowledges the dynamic nature of biocultural diversity, (e.g., through the appropriation of introduced species and new varieties into the local knowledge system, practices, and worldviews in Hilgert et al. 2014 or Villalba and Lambaré 2019), along with the links between biocultural diversity and food sovereignty (Gutiérrez Escobar 2019). Regarding research methods, participatory approaches are proposed as suitable to assess biocultural diversity (Aldasoro Maya and Argueta Villamar 2013).

#### Biocultural heritage, knowledge, and memory

This lens considers biocultural heritage as the result of a long interaction between cultures and nature (Socies Fiol and Cuéllar Padilla 2017; Ruiz Barajas 2018). Biocultural heritage is usually connected to specific places or territories (Hernández Hernández et al. 2018; García Flores 2020; Mathiesen 2020) and is described as encompassing beliefs, knowledges, and practices—or cosmos, corpus, and praxis (as in Vásquez González et al. 2016). This understanding of heritage closely relates to conceptualizations of biocultural knowledge and memory (as in Toledo and Barrera-Bassols 2008). This lens includes empirical studies that describe specific types of knowledge. Illustrative cases are the analysis of biocultural knowledge with regard to agrobiodiversity (Socies Fiol and Cuéllar Padilla 2017; Marchant Santiago et al. 2020), traditional medicine (Hirose López 2018; Scarpa and Rosso 2010; Correo and Pirondo 2019), knowledge of bird and fungus species and biocultural memory (Nuñez-García et al. 2012; Padilla-Mejia and Ramírez Calvo 2019; Montoya et al. 2019), and knowledge and heritage related to rituals or festivities and culinary traditions (Espinoza López et al. 2016; Vásquez González et al. 2016, Santos Tanús et al. 2019; Bañuelos-Flores and Salido-Araiza 2020; Martínez et al. 2020). Studies within this lens also pay attention to the role of language and oral traditions in biocultural knowledge transmission pathways and to the dynamics of continuity and erosion (Cupul Cicero et al. 2019; Montoya et al. 2019). Particularly, this lens emphasize that biocultural heritage, conservation, and development are tightly linked (Guzmán 2016; Avellaneda-Torres et al. 2014; Hernández Hernández et al. 2018; García et al. 2019; Diaz et al. 2021). The maintenance and revitalization of biocultural knowledge and heritage is seen as a prerequisite for the defense of territories (Avellaneda-Torres et al. 2014; Ruiz Barajas 2018; Román Suárez et al. 2021) and to achieving a diverse and just society (Costanzo 2016). Towards this goal, Eguiarte Espejo (2017) brings to the fore the role of activism to strengthen identity and biocultural heritage through the arts (“artivism”).

#### Biocultural conservation lens

This lens promotes an alternative model to conventional biodiversity conservation (Torrealba and Carbonell 2008; Cordero Romero and Palacio 2018) supported by the premise that biodiversity cannot be preserved without protecting cultural diversity (Rodríguez-Ramírez et al. 2017). Conventional conservation is interpreted in many articles as an external model that neglects local people’s needs and perspectives about nature (Neira Brito 2006; Torrealba and Carbonell 2008; Torrescano Valle et al. 2018), which is seen as raising ethical and justice issues (Nemogá 2016). Examples of biocultural conservation include those that explicitly focus on the conservation of biocultural diversity (Neira Brito 2006; Bartl 2019), those that consider the participatory management and monitoring of biodiversity (La Torre-Cuadros 2013; Doumecq et al. 2020; Maldonado Ibarra et al. 2020) as well as those that highlight the relevance of traditional practices for biodiversity conservation (Martínez González and López-Prado 2014; Cuevas Coeto et al. 2019; Mastretta-Yanes et al. 2019; Montaño et al. 2021). Overall, the idea is that biocultural conservation fundamentally changes the underlying principles of conservation (Rozzi et al. 2013, 2020), implying transformations in research and education (Rozzi and Schüttler 2015; Nemogá 2016). Furthermore, the need to care for biocultural diversity is seen as a prerequisite for an alternative model of conservation and development (Torrealba and Carbonell 2008), especially one that is endogenous or locally embedded (Sánchez-Zárate 2016; Figueroa Burdiles and Vergara-Pinto 2018).

#### Biocultural ethics

Articles in this lens emphasize that environmental ethics should be the foundation of the biocultural paradigm for the conservation of biological and cultural diversity (Toledo 2013; Rozzi 2016). Conceptual contributions in this lens argue about the mutual determination of habitats, inhabitants (humans and non-humans), and their habits in every ecosystem or culture (Céspedes 2018). Such locally embedded environmental philosophy and ethics is considered as being the basis for integrating biocultural research, conservation, and education (Rozzi 2018). Several articles illustrate this through case studies. These case studies draw upon the methodological approach of field environmental philosophy, which includes biophysical-ecological and philosophic aspects of research, communication through metaphors and narratives, the design of field experiences and the implementation of *in-situ* protected areas (Rozzi et al. 2010). Examples include educational and ecotourism activities to engage and connect with bryophytes and lichens, living beings that are generally under-perceived and valued (Lewis et al. 2018), field workshops to encounter with freshwater invertebrates to fostering perceptions of either their intrinsic value and their value as climate change bioindicators (Contador et al. 2018), as well as studies that integrate scientific and local understandings of invasive species through metaphors (Crego et al. 2018). Relational values are also proposed as a way to incorporate biocultural dimensions into biodiversity research and as indicators for the evaluation of environmental management projects (Barreto and Redón 2020, for the case of cultivation of native bee species).

#### Biocultural landscape lens

Articles in this lens describe landscapes as sociocultural constructions with an emphasis in the spatial manifestation of the interactions between societies and their territories (Oases in Lower California in Olivera and Maldonado 2017). Biocultural or cultural landscapes studies tend to take a historical and heritage perspective (Mancera-Valencia 2019; Montoya and Toledo 2020). This includes historical transformations of biocultural landscapes related to the Spanish colonization in the Americas (Ojeda 2019), along with investigations of the role of biological diversity and its management for heritage within urban spaces (Cuvi 2017). Besides historical and heritage aspects, the importance of local worldviews and spiritual dimensions in biocultural landscapes are highlighted (Gonzales 2017).

#### Biocultural education

This lens places the need of a decolonization of epistemologies at the foreground, and thus the importance of incorporating biocultural memory and heritage into school and universities’ curricula (Bravo Osorio 2016; Mancera-Valencia et al. 2018; Pacheco-Calderón 2019) as well as in extracurricular and non-formal activities of environmental education (García Campos 2013; Malebrán and Rozzi 2018; Vázquez 2019; Medina et al 2020). Especially, the idea of a practical pedagogy is often mentioned (Bravo Osorio 2016; Malebrán and Rozzi 2018; Medina et al. 2020) as a way to reinforce and revitalize people’s connectedness to their territories and the conservation of biocultural diversity. While articles in this lens usually aim to reshape educational and pedagogical practices, links are also made to the potential implications of these for broader societal transformations. Authors like Malebrán and Rozzi (2018) and Medina et al. (2020) contend that biocultural education can contribute to reconnect and increase empathy of humans with non-human beings through environmental philosophy field courses and ecotourism.

#### Biocultural tourism

Within this lens, biocultural tourism is presented as an activity that can complement local peoples’ livelihoods by supporting the revalorization and continuity of traditional practices, biocultural heritage and rights over the territories (Torres Villa and López 2016; Cervantes and Serrano 2017; Thomé-Ortiz and García-Soto 2019). The articles in this lens draw on the idea of endogenous development, i.e., emanating from local priorities, needs, and values (Jiménez Ruiz et al. 2016; Jasso Arriaga 2017; Thomé-Ortiz and García-Soto 2019; Cervantes and Serrano 2019). Biocultural tourism is seen as an alternative development pathway that focuses primarily on fostering reciprocity, respect and empathy, instead of only economic revenues (Cervantes and Serrano 2017). Often the ambivalence between the benefits and the potential threats of tourism – for example, tourist overcrowding—are also highlighted and criteria for the implementation of biocultural tourism are suggested (Cervantes and Serrano 2017; Jasso Arriaga 2017; Ángel et al. 2020).

#### Biocultural ontologies and epistemologies

Articles within this lens point out the multiple ways of thinking, living and doing that differ from the modern or Eurocentric paradigm. The biocultural perspective is applied as a framework that allows the acknowledgment and revalorization of ontologies and epistemologies that emerged in the global South and that have been historically excluded (Mariaca 2019; Rossetti 2019). The ontological and political proposal of *Vivir bien* o *Buen Vivir* from Aymara and Quechua Andean Indigenous Peoples is the focus of some of the contributions to this lens (Arguello and Cueva 2009; Rosetti 2019). A decolonial inquiry praxis based on knowledge dialogs (*diálogos de saberes*) and transdisciplinarity are generally employed in empirical studies (Arguello and Cueva 2009; Pérez-Mesa 2019; Aiterwegmair et al. 2021; Salas and Tillmann 2021), besides ethnographic and descriptive studies (Rossetti 2019). Agriculture and agroecology are considered to be examples of biocultural systems or complexes of reciprocity between human societies and nature, illustrated by the case of Andean and Maya cosmovisions (Arguello and Cueva 2009; Luque et al. 2012a, b; Mariaca 2019). Epistemic or cognitive justice is an underlying motivation for articles in this lens (Aiterwegmair et al. 2021; Salas and Tillmann 2021).

#### Biocultural rights and nature’s rights

This lens looks at different types of rights that reflect an intercultural understanding of justice and the socioenvironmental conflicts derived from the multiplicity of visions of ecological ethics (Medici 2018; Caguana and Naranjo 2020; Olvera 2020). The notion of biocultural rights contemplates the relational interdependence between nature and culture, that is, between Indigenous peoples and local communities’ lifeways and their territories. The recognition and protection of biocultural rights and biocultural heritage are one of the themes of articles within this lens (Patrick-Encina and Bastida Muñoz 2010; Domínguez 2020; Millaleo Hernández 2020). Scholarship in this lens also explores innovations in law and jurisprudence in many countries and regions worldwide in expanding the anthropocentric conception of law to the recognition and protection of nature on the basis of its own rights and intrinsic value, granting legal personhood to natural entities (e.g., rivers, moorlands, glaciers) towards a new paradigm of ecocentric justice (Cagüeñas et al. 2020; Canagua and Naranjo 2020; Millaleo Hernández 2020; Giménez 2020) also known as *Earth Jurisprudence* (see Medici 2018). In some cases, the recognition of legal rights to nature resolves historical claims of Indigenous peoples (Giménez 2020). For some authors, this also presents an opportunity for decolonization (Caguana and Naranjo 2020; López-Barreto 2021) and for extending the scenarios of reparation of nature beyond those places that are inhabited, in order to break the narratives that sustain practices of structural violence towards nature (Ramírez-Hernández and Leguizamos-Arias 2020). For Millaleo Hernández (2020), biocultural rights and nature rights are closely related and complementary in viewing Indigenous peoples as nature stewards. Moreover, within this lens the role of biocultural protocols in assisting the implementation of the Nagoya protocol and avoiding biopiracy is also explored (Hernández Ordoñez 2019).

### Comparison of biocultural lenses

**Fig. 3:**
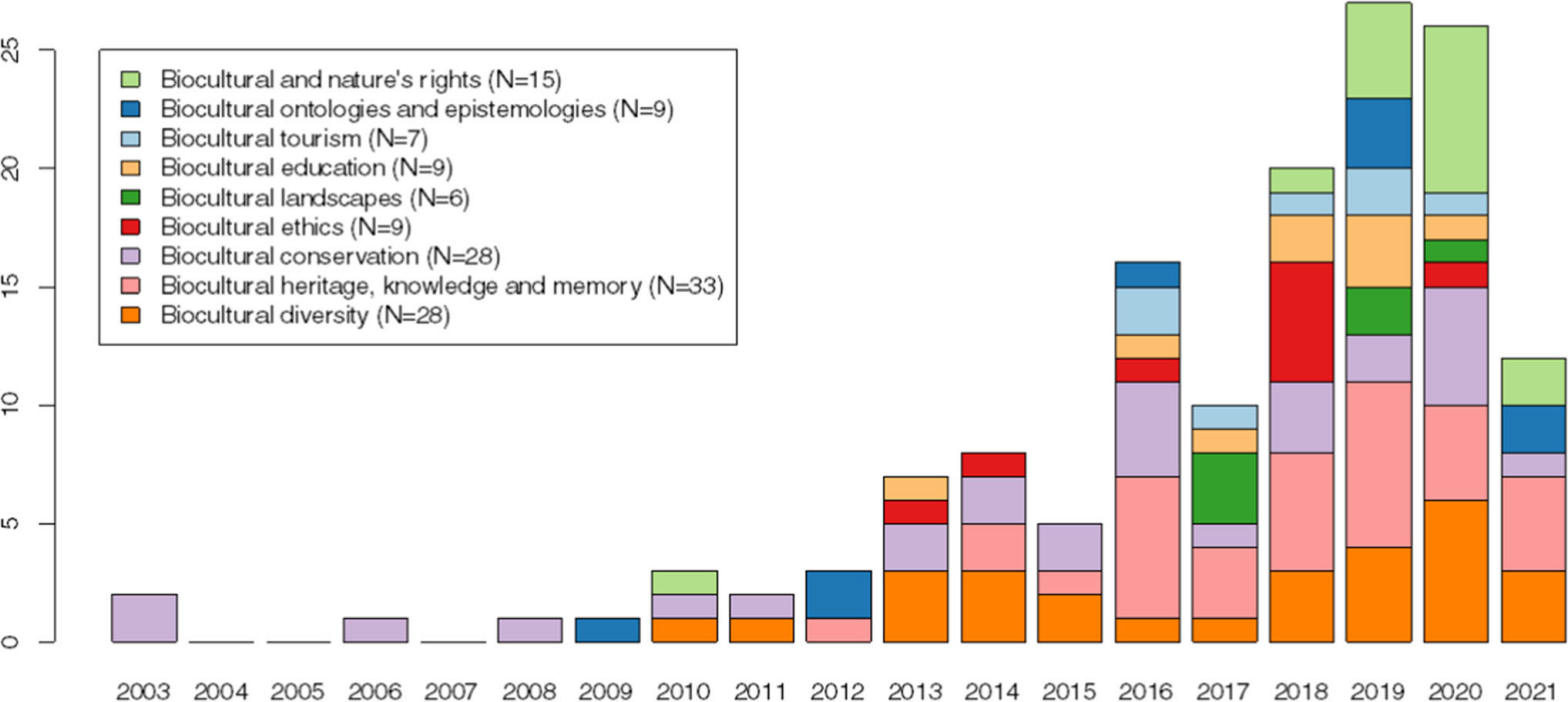
Frequency of articles and distribution of emergent primary lenses by year. The collection of bibliometric data for this review was done in October 2021; thus, it does not capture the full set of papers published in 2021

The frequency of articles and distribution of the emergent lenses over time (Fig. 3) display a relatively steady increase in the number of articles since 2010, with 2019 and 2020 being especially productive for biocultural approaches in the academic literature in Spanish reviewed. The overall increase in the number of articles over time is associated with a general diversification in lenses although the different lenses are not equally represented in the literature. For example, biocultural heritage, knowledge and memory (*N* = 33), biocultural diversity (*N* = 28), and biocultural conservation (*N* = 28) are by far the most frequently represented lenses. The biocultural heritage, knowledge, and memory lens are present in over five times as many articles as the least-frequent lenses biocultural tourism (*N* = 7) and biocultural landscapes (*N* = 6). The biocultural rights and nature’s rights lens (*N* = 15) increased quite strongly in recent years.

**Fig. 4:**
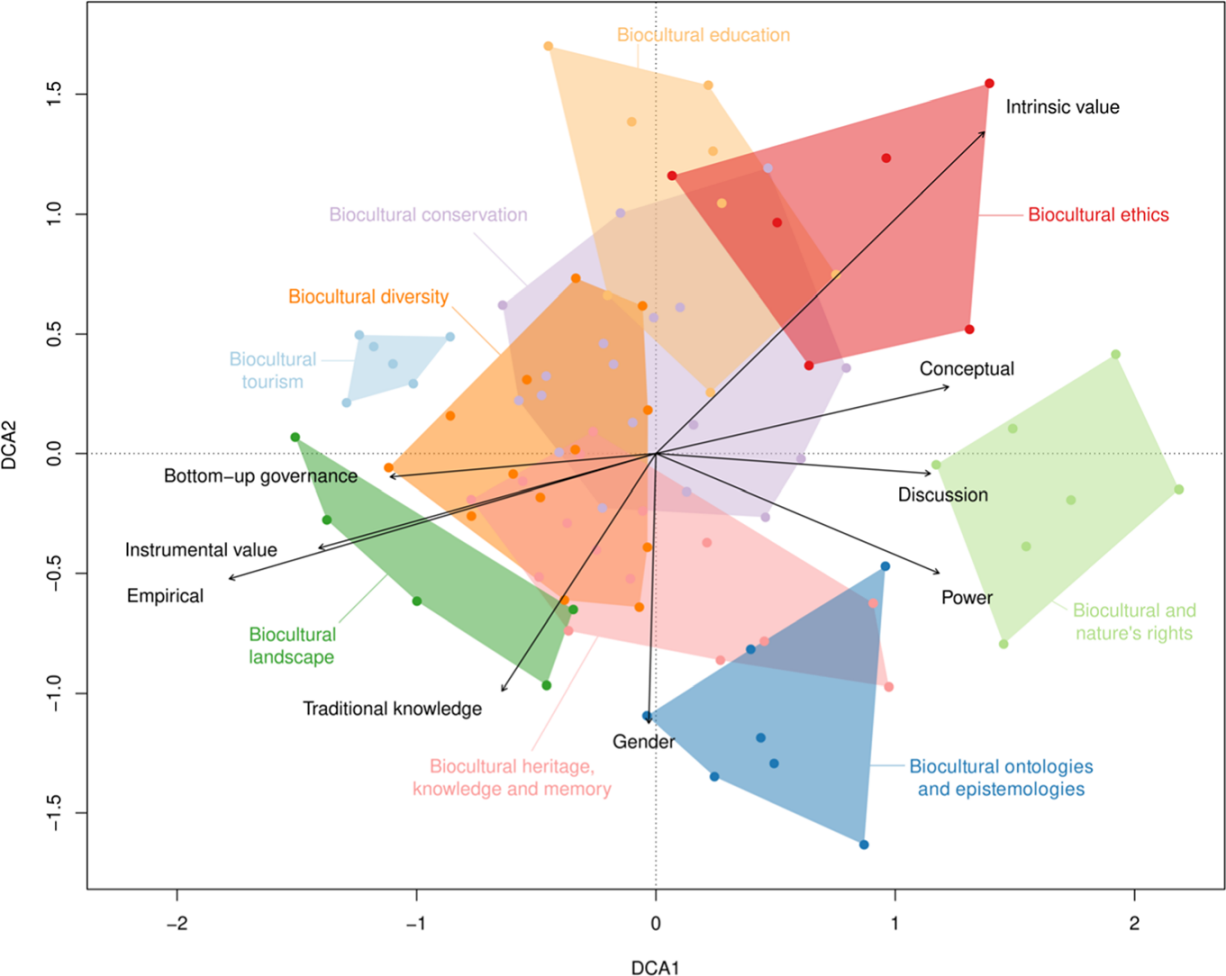
Results of the detrended correspondence analysis (DCA) of the primary and secondary lenses of the papers for the first two axes. Dots represent individual papers and polygons enclose all papers belonging to a similar primary lens (colored labels). Arrows indicate characteristics of the papers that were significantly correlated to the ordination space (see Table 1 for a description of the characteristics of the papers: For Empirical, Conceptual and Discussion see Type of paper; for Intrinsic value and Instrumental value see Value type; for Traditional knowledge see Knowledge type; and for Bottom-up governance see Governance). Items close to each other are very similar and distant items very dissimilar to each other

The multivariate analysis of the lenses showed that the biocultural conservation, the biocultural diversity, and the biocultural heritage, knowledge, and memory lenses are very central to the ordination diagram (Fig. 4, First axis eigenvalue 0.51, length of first axis 3.7; Second axis eigenvalue 0.42, length of second axis 3.3. See also Table S1 in Supplementary information). This indicates not only that a large proportion of the articles reviewed correspond to these three lenses as a primary lens, but also that many other articles include ideas related to these lenses as secondary lens. Bottom-up governance also appears strongly related to the biocultural diversity lens. Gender and traditional knowledge variables appear strongly correlated with each other and with the biocultural heritage, knowledge and memory lens. On the right-hand side of the ordination plot, there are mainly articles from the biocultural ethics, the biocultural rights, and the biocultural ontologies and epistemologies lenses. Articles on this side tend to be conceptual or discussions and consider intrinsic values of biocultural diversity (biocultural ethics) and power (biocultural rights and biocultural ontologies and epistemologies). On the left-hand side of the diagram, there are the biocultural diversity, and the biocultural landscapes lenses. Articles on this side tend to be empirical and emphasize traditional and local knowledge, and the instrumental value of biocultural diversity. More marginal to the ordination diagram are the biocultural ontologies and epistemologies lens, the biocultural tourism, and the biocultural education lenses.

## Discussion

This review indicates that biocultural approaches in the scientific literature in Spanish are conceptually diverse and complex, a meeting place of distinctive understandings of “biocultural.” In a nutshell, some lenses primarily focus on describing how nature and culture are co-produced (in lenses of biocultural diversity, landscapes, knowledge, heritage, and memory) and others suggest actions in relation to biocultural diversity (in conservation, rights, ethics, education, tourism, ontologies, and epistemologies lenses).

We can highlight some convergent findings between the academic literatures on biocultural approaches to sustainability in English and Spanish. For one, issues related to knowledge, heritage, and memory of biocultural diversity are most prominent also in English-language literature (Bridgewater and Rotherdam 2019; Hanspach et al. 2020). Biocultural approaches to biodiversity conservation are also seen as instrumental to achieving more environmentally just conservation outcomes by taking into account values beyond the intrinsic value of biodiversity, and by including the priorities and needs of local people (Gavin et al. 2015).

Besides that, there are innovative aspects in the biocultural approaches in the literature in Spanish compared to those in the literature in English. We have identified three novel emergent lenses: biocultural tourism, biocultural education, and biocultural ontologies and epistemologies. The biocultural tourism lens links to alternative development models and initiatives that are endogenous and probably reflect the growing importance of “ethnic” tourism in Latin America (Sánchez-Zárate 2016; Cervantes and Serrano 2017; Figueroa Burdiles and Vergara-Pinto 2018), and highlights the revalorization and continuity of the diversity of lifeways and peoples. The biocultural education lens invites one to rethink education in a way that allows the emerging local epistemologies and ontologies to flourish, and thus, is based in a pedagogy nurtured by intercultural dialogs and knowledge dialogs towards a pluricultural paradigm of biocultural heritage management (Mancera-Valencia 2018; see also de Tattay 2013; Rivera et al. 2017). The biocultural education lens also reflects the appearance of Indigenous and intercultural universities and educational centers in Latin America and underlines the significance of non-formal and informal education (Vázquez 2019). The biocultural ontologies and epistemologies lens presents a vindication of the epistemologies of the South (Sousa Santos 2018; Alarcón-Cháires 2017) and of the longstanding ontological and relational perspectives in Latin America’s environmental activism and transdisciplinary social-ecological research (Escobar 2015). These novel lenses could indeed be bridged to the biocultural “transformations” lens identified by Hanspach et al. 2020 in the English literature. The literature in Spanish provides complementary ideas of societal change from alternative development paradigms, including alternative endogenous pathways, knowledge dialogs (*diálogos de saberes*), and the importance of epistemic justice, which could be seen as “emancipatory transformations” of human-nature relationships (Sánchez-Zárate 2016; Cervantes and Serrano 2017; Figueroa Burdiles and Vergara-Pinto 2018; Aiterwegmair et al. 2021; Salas and Tillmann 2021). Lastly, while Hanspach et al. (2020) also identified issues related to biocultural rights in the English literature, this review brings new insights into how biocultural approaches in Spanish are taking on the relatively recent development of Nature rights in Latin America and beyond (Caguana and Naranjo 2020; Millaleo Hernández 2020; López-Barreto 2021). The recognition of nature’s rights constitutes a legislation revolution beyond traditional western law. However, the interplay between biocultural rights and nature’s rights and its consequences are, for some cases, still into question. For example, some evidence suggests that granting legal rights to non-human beings may gloss over the rights of Indigenous peoples and their role in nature governance (Macpherson et al. 2020). This may be the case when biocultural rights and the role of Indigenous peoples and local communities as nature stewards according to their culture are overlooked by the courts providing legal rights to nature (Macpherson et al. 2020). Therefore, this opens an arena for further inquiry within biocultural approaches to sustainability.

Along with these new perspectives, there are additional complementarities that can support further developing biocultural research in the scientific literature in English. Hanspach et al. (2020) showed that social sustainability and justice have not been extensively considered in the application of biocultural approaches in the literature in English thus far. Our findings reveal that the consideration of power is more prevalent in the Spanish review, where it was taken into account in more than half of the articles reviewed. Specifically, power is more prominent in the biocultural rights and nature’s rights as well as biocultural ontologies and epistemologies lenses. This consideration offers counter-hegemonic and decolonizing perspectives for the conceptualizations and applications of biocultural approaches more generally. For example, knowledge dialogs and transdisciplinary methods that explicitly acknowledge and address power issues (Arguello and Cueva 2009; Aiterwegmair et al. 2021; Salas and Tillmann 2021; see also Turnhout et al. 2020) could counter some of the caveats highlighted in Hanspach et al. (2020). These findings also match well with Merçon et al. (2019) in underscoring the importance of the biocultural paradigm for Indigenous rights and civil society movements in Latin America fighting neo-colonial power dynamics and advocating for social-ecological justice.

Our review also points out that gender issues are overlooked to a great extent in the biocultural approaches in the literature in Spanish, a gap that was also noted in Hanspach et al. (2020). In both literatures, gender is considered in relation to gendered types of knowledge. This result underlines that the gender bias evident in environmental and sustainability scientific research (Howard 2003; Ravera et al. 2016) applies also to biocultural research. Other voids that have potential for improvement are the engagement with knowledge holders and stakeholders in a genuine participatory manner—since our review, and also the one by Hanspach et al. (2020), highlight that the inclusion of non-academic actors is generally made only by consultation. Additionally, there seems to be a tendency towards centering mainly on the cultural aspects of the biocultural, leaving pace for a better inclusion of ecological or environmental aspects.

We also see future research pathways and collaborations between the Spanish and English-speaking academic worlds. Biocultural approaches to sustainability in more dynamic systems with a broader type of communities (e.g., in urban areas and beyond the Indigenous and local, Cocks 2006) seem to be a promising avenue to better grasp transformations in the rural–urban continuum, especially in Latin America, in relation to environmental justice and ontological conflicts in ways of understanding the territories and cities (Cocks et al. 2020; McMillen et al. 2020; Stålhammar and Brink 2021). In this sense, biocultural approaches to territorial and environmental governance through local, endogenous processes that are rooted in the rights, knowledge, customary practices, and identities of those who remain stewards of biocultural diversity could support the thriving of diverse places and territories (Apgar 2011, 2017; Gavin et al. 2018; Hernández-Hernández y Llanos-Hernández 2019; López-Barreto 2021). Likewise, gender intersectional perspectives that recognize the context-specific social differentiation and inequality axes, such as age or ethnicity, among many others, in the access, use, management, and control of biodiversity as well as in the understandings of human-nature relationships, would provide a more robust basis to biocultural studies. Moreover, relational values are prominent both the Spanish and the English biocultural literature (Merçon et al. 2019; Hanspach et al 2020) highlighting the importance of meaningful relationships, care, and stewardship (Chan et al. 2016; Stålhammar and Brink 2021), which also provides opportunities to consider different ways of assessing emerging values from human-nature relations. In particular, decolonial epistemological perspectives can open innovative methodological pathways in this direction. For example, knowledge dialogs, transdisciplinary, and participatory action research, can bring together diverse actors from social movements and academia with their plurality of ontologies and epistemologies in considering biocultural relations (Alarcón-Cháires 2017; Reiter 2018; Sousa Santos 2018; Escobar 2020).

## Conclusions

This article highlights the contributions of the scientific literature in Spanish, which is to a great extent a Latin America perspective, to the biocultural paradigm (sensu Merçon et al. 2019). Our results reveal the richness of biocultural approaches in contexts beyond the Anglophone predominance in academic knowledge production and communication. They also reveal that decolonial methodological pathways can contribute more epistemic and environmentally just biocultural research that supports emancipatory transformations of human-nature relationships. For example, by acknowledging and addressing power relations through dialogs with other knowledges systems, which have the potential to nurture creative endogenous processes by weighting relational values of biocultural diversity. Nevertheless, these are just initial steps, considering that, for example, most Indigenous and local knowledge is produced and transmitted in diverse ways other than academic and scientific works. Also, research could take a historical perspective to explore the likely co-evolution and mutual influence of publications in English and Spanish language. Biocultural approaches to sustainability in the literature in Spanish could be also enriched by addressing the intersectional aspects of gender for new insights both for research and practice. Studies could further integrate perspectives from more dynamic and transformed systems (i.e., beyond Indigenous and local communities). Lastly, inquiry into the interplay between biocultural rights and nature’s rights and its challenges and opportunities for Indigenous People’s nature stewardship as well as involvement in and control over their territories might be a future pathway for the development of biocultural research.

### Supplementary Information

Below is the link to the electronic supplementary material.

Supplementary file1 (PDF 974 kb)
