# Supplementary material for: Appraising biocultural approaches to sustainability in the scientific literature in Spanish
Source: Ambio. 2024 Jan 24;53(4):499–516. doi: 10.1007/s13280-023-01969-3 (PMC10920613; doi:10.1007/s13280-023-01969-3)
Supplement: Supplementary file 1 — Supplementary file1 (PDF 974 kb) [file 13280_2023_1969_MOESM1_ESM.pdf]

Ambio

Electronic Supplementary Information

This supplementary information has not been peer reviewed.

Title: **Appraising biocultural approaches to sustainability in the scientific literature in Spanish**

Authors: Isabel Díaz-Reviriego, Jan Hanspach, Mario Torralba, Stefan Ortiz Przychodzka, Camila Benavides Frias, Leonie Burke, María García-Martín, Elisa Oteros-Rozas

Appendix S1

List of papers reviewed

Aiterwegmair, K. *et al.* Recreando agri-cultura: Conocimientos agroecológicos e identidades campesinas en un proceso de educación-investigación-acción en Chiapas, México. *Revista Brasileira de Educação do Campo* **6,** 1–30 (2021).

Aldasoro Maya, E. M. & Argueta Villamar, A. Colecciones etnoentomológicas comunitarias: una propuesta conceptual y metodológica. *Etnobiología* **11,** 1–15 (2013).

Álvarez Gordillo, Guadalupe del Carmen, Vera Cortés, G. & Ramos Muñoz, D. E. Vulnerabilidad y patrimonio biocultural en Tacotalpa, Tabasco. *Política y Cultura* **45,** 211–239 (2016).

Arguello, M. & Cueva, K. La revalorización de la agroecología andina: Estrategia local de diálogo de saberes para enfrentar problemas globales. *Letras Verdes, Revista Latinoamericana de Estudios Socioambientales* **12,** 12–15 (2009).

Argueta Villamar, A. El estudio etnobioecológico de los tianguis y mercados en México. *Revista Etnobiologia.* **14,** 38–46 (2016).

Avellaneda-Torres, L. M., Rojas, E. T. & Sicard, T. E. L. Alternativas ante el conflicto entre autoridades ambientales y habitantes de áreas protegidas en páramos colombianos. *Mundo Agrario* **16** (2015).

Avellaneda-Torres, L. M., Torres Rojas, E. & León Sicard, T. E. Agricultura y vida en el páramo: Una mirada desde la vereda El Bosque (Parque Nacional Natural de Los Nevados). *Cuadernos de Desarrollo Rural* **11,** 105–128 (2014).

Bañuelos-Flores, N. & Salido-Araiza, P. L. Enredados con la sierra. Las plantas en las estrategias sostenibles de sobrevivencia del grupo indígena Guarijío/Makurawe de Sonora, México. *Revista Tecnología en Marcha* **33,** 178–192 (2020).

Bartl, B. 'La yaquispala': Prácticas Locales, Memoria Y conservación. *Boletín De La Sociedad Argentina De Botánica* **54,** 451–471 (2019).

Bello Cervantes, I. Montserrat Pérez Serrano, A. Turismo y preservación biocultural en la Matlalcuéyetl. Caso San Pedro Tlalcuapan, Tlaxcala, México. *Regiones y Desarrollo Sustentable* **19,** 109–136 (2019).

Bello Cervantes, I. & Pérez Serrano, A. M. Turismo biocultural: relación entre el patrimonio biocultural y el fenómeno turístico. Experiencias investigativas. *Scripta Ethnologica* **39,** 109–128 (2017).

Bernis Carro, C. Integrando formación e investigación sobre la transversalidad de los ODS: Género y salud, dimensiones esenciales de la sostenibilidad. *Ciencia, Técnica y Mainstreaming Social* **3,** 1–12 (2019).

Bravo Osorio, L. M. Escuela, memoria biocultural y territorio: el caso de la práctica pedagógica integral en la institución educativa Inga Yachaikury (Caquetá-Colombia). *Revista Educación y Ciudad* **30,** 159–166 (2016).

Cababié, J., Bonicatto, M. M. & Abbona, E. A. Semillas y saberes de los agricultores familiares. ¿Cuál es el rol de las ferias de intercambio en su reproducción y conservación? *Revista de la Facultad de Agronomía* **114,** 122–128 (2015).

Cagüeñas, D., Galindo Orrego, M. I. & Rasmussen, S. El Atrato y sus guardianes: imaginación ecopolítica para hilar nuevos derechos. *Revista Colombiana de Antropología* **56,** 169–196 (2020).

Califano, L. M. Gestión del pastoreo: conocimientos y prácticas de manejo de las especies forrajeras en la ganadería trashumante de Iruya (Salta, Argentina). *Boletín De La Sociedad Argentina De Botánica* **55,** 493–513 (2020).

Califano, L. M. Percepción y manejo del paisaje y de los recursos vegetales por campesinos trashumantes de Iruya (Salta, Argentina). *Bonplandia* **29,** 101–118 (2020).

Calvet Mir, L., Garnatje Roca, T., Vallès Xirau, J. & Reyes-García, V. Más allá de la producción de alimentos: Los huertos familiares como reservorios de diversidad biocultural. *Ambienta: La revista del Ministerio de Medio Ambiente* **107,** 40–53 (2014).

Campregher, C. Conservación de la diversidad bio-cultural en Costa Rica: comunidades indígenas y el ambiente. *Cuadernos de Antropología* **21,** 1–20 (2011).

Castello, A. P., Hurrell, J. A. & Pochettino, M. L. Estrategias metodológicas para acceder a la diversidad biocultural en huertos del periurbano platense (Buenos Aires, Argentina). *Bonplandia* **30,** 5–26 (2021).

Céspedes, E. Propiedades emergentes, valor intrínseco y ecología: Algunas perspectivas éticas sobre los habitantes de la isla navarino. *Magallania* **46,** 227–235 (2018).

Chan Mutu , Guelmy Anilú, Elda Miriam, M. M. & Sotelo Santos, E. L. Retomando saberes contemporáneos. Un análisis del panorama actual de la meliponicultura en Tabasco. *Estudios de Cultura Maya* **53,** 287–326 (2019).

Colin-Bahena, H., Monroy, R., Velázquez-Carreño, H., Velázquez-Carreño, A. & Monroy-Ortiz, C. El tianguis de Coatetelco, Morelos: articulador de la conservación biocultural en el territorio. *Revista Etnobiologia* **16,** 87–97 (2018).

Concha , Roberto Gabriel Mathiesen. Patrimonio cultural inmaterial y sustrato biocultural en paisajes de San Pedro de Atacama, Región de Antofagasta y Río Ibáñez, Región de Aysén. *Revista Pensamiento y Acción Interdisciplinaria* **6,** 31–49 (2020).

Contador, T. *et al.* Sumergidos con lupa en los ríos del cabo de hornos: Valoración ética de los ecosistemas dulceacuícolas y sus co-habitantes. *Magallania* **46,** 183–206 (2018).

Cordero Romero, S. S. & Palacio, G. Parques Nacionales desde la percepción local: A propósito del Parque Nacional Natural Amacayacu (Amazonas, Colombia). *Mundo Amazónico* **9,** 199–227 (2018).

Cortés, J., Ugalde, I., Caviedes, J. & Ibarra, J. T. Semillas de montaña recolección, usos y comercialización del piñón de la araucaria (Araucaria araucana) por comunidades Mapuche-Pewenche del sur de los Andes. *Pirineos* **174** (2019).

Costanzo, M. Perspectivas de cambio desde el Sur. Pensamiento crítico desde la raíz. *Cuadernos de Filosofía Latinoamericana* **37,** 45–69 (2017).

Cotler-Ávalos, H. & Lazos-Chavero, E. La multifuncionalidad de agroecosistemas en la cuenca del río Cuitzmala, Jalisco, México. *Agricultura Sociedad y Desarrollo* **16,** 513–537 (2020).

Crego, R. D., Ward, N., Jiménez, J. E., Massardo, F. & Rozzi, R. Los ojos del árbol: percibiendo, registrando, comprendiendo y contrarrestando las invasiones biológicas en tiempos de rápida homogeneizacion biocultural. *Magallania* **46,** 137–153 (2018).

Cruz Vázquez, M. Recursos bioculturales para la alimentación de las comunidades indígenas de la Sierra del Totonacapan Veracruzano. *Interconectando Saberes* **4,** 173-183 (2019).

Cuevas Coeto, A., Vera Castillo, Y. B. & Cuevas Sánchez, J. A. Resiliencia y sostenibilidad de agroecosistemas tradicionales de México: Totonacapan. *Revista Mexicana de Ciencias Agrícolas* **10,** 165–175 (2019).

Cupul Cicero, V., Aguilar Cordero, W. D. J., Chablé Santos, J. & Sélem Salas, C. I. Conocimiento etnozoológico de la herpetofauna de la comunidad maya de Santa Elena, Yucatán, México. *Estudios de Cultura Maya* **54,** 285–314 (2019).

Cuvi, N. Las ciudades como mosaicos bioculturales: El caso del centro histórico de Quito. *Revista Etnobiologia* **15,** 5–25 (2017).

D'Alessandro Nogueira, R. & González Cabañas, A. A. Siete controversias capitales: análisis de la implementación del Programa Maíz Solidario en Los Altos de Chiapas. *LiminaR Estudios Sociales y Humanísticos* **12,** 129–147 (2014).

Doumecq, M. B., Petrucci, N. S. & Stampella, P. C. Cuando los saberes no dialogan. Prácticas en conflicto en el Parque Costero Sur (Buenos Aires). *Bonplandia* **29,** 57–70 (2020).

Eguiarte Espejo, C. P. Corporalidad, creación escénica y teatro comunitario en la Universidad Veracruzana Intercultural (UVI). *Entreciencias: Diálogos en la Sociedad del Conocimiento* **5** (2017).

Espinoza López, P. C., Bañuelos Flores, N. & López Reyes, M. Entre capullos de mariposas y fiestas. Hacia una alternativa de turismo indígena en El Júpare, Sonora, México. *Estudios sociales* **24,** 312–344 (2016).

Figueroa Burdiles, N. & Vergara-Pinto, F. Reserva Nacional China Muerta: Consideraciones en torno a la conservación biocultural de la naturaleza, los incendios forestales y la herida colonial en territorios indígenas. *CULTURA-HOMBRE-SOCIEDAD* **28,** 102–127 (2018).

García Campos, H. M. La educación ambiental con enfoque intercultural. Atisbos latinoamericanos. *Revista Biografía: Escritos sobre la biología y su enseñanza* **6,** 161–168 (2013).

García Flores, A. Ethnoecological study of the birds of Coatetelco, Morelos, Mexico. *Ecosistemas* **29,** 1–10 (2020).

Garrido Peña, F. Topofilia, paisaje y sostenibilidad del territorio. *Enrahonar. Quaderns de filosofia* **53,** 63–75 (2014).

Gonzales, T. Turismo, cocinas, sabores y saberes locales y regionales sostenibles en Perú. *Turismo y Patrimonio* **11,** 37–51 (2017).

Guadarrama Martínez, N., Chávez Mejía, M. C., Rubí Arriaga, M. & White Olascoaga, L. La diversidad biocultural de frutales en huertos familiares de San Andrés Nicolás Bravo, Malinalco, México. *Sociedad y Ambiente,* 237–264 (2020).

Guarino, G. B. & Pirondo, A. Patrimonio biocultural y resiliencia en los pueblos indígenas del Chaco, Argentina. *Inventio* **15,** 33–43 (2019).

Gutiérrez Escobar, L. Diversidad biocultural, agricultura raizal y soberanía alimentaria en San Andrés y Providencia (Colombia). *Tabula Rasa* **32,** 195–225 (2019).

Guzmán, D. Diversidad biocultural y género: Trayectorias productivas de mujeres campesinas de Chiloé. *Revista Austral de Ciencias Sociales* **31,** 25–42 (2016).

Hernández Bernal, M. C. Los alimentos en la vida ritual de los nahuas de San Juan Tetelcingo, Guerrero. Un elemento a considerar dentro del patrimonio biocultural. *Dimensión AntropológicA* **23,** 64–86 (2016).

Hernández Hernández, B. R., Santiago Ibañez, D. P., Miguel Velasco, A. E., Cruz Carrasco, C. & Regino Maldonado, J. Empresas sociales rurales, estrategía de desarrollo sustentable y conservación del patrimonio cultural inmaterial: Caso: Amaranto (Amaranthus spp) de Mesoamerica. *Revista Mexicana de Agronegocios* **42,** 955–967 (2018).

Hernández Ordoñez, S. R. El protocolo de Nagoya en México: un análisis legal del cumplimiento y el papel de los protocolos comunitarios bioculturales. *Revista de la Facultad de Derecho de México* **69,** 611–646 (2019).

Hernández-Hernández, A. & Llanos-Hernández, L. La reconfiguración y apropiación del territorio agrícola en Ixcanelco a través de las prácticas socioculturales. *Revista de Geografía Agrícola* **62,** 9–27 (2019).

Hilgert, N., Lambare, A. D., Vignale, N. D., Stampella, P. C. & Pochettino, M. L. ¿Especies naturalizadas o antropizadas? Apropiación local y la construcción de saberes sobre los frutales introducidos en época histórica en el norte de Argentina. *Revista Biodiversidad Neotropical* **4,** 69–87 (2014).

Hirose López, J. La medicina tradicional maya: ¿Un saber en extinción? *Revista Trace* **74,** 114–134 (2018).

Isabel Moreno-Calles, A., M. Toledo, V. & Casas, A. Los sistemas agroforestales tradicionales de México: Una aproximación biocultural. Una aproximación biocultural. *Botanical Sciences* **91,** 375–398 (2014).

Jiménez Ruiz, A., Thomé-Ortiz, H. & Burrola-Aguilar, C. Patrimonio biocultural, turismo micológico y etnoconocimiento. *El Periplo Sustentable* **30,** 180–205 (2016).

La Rorsa, E. P. de, Hernández Cuevas, F. I., Castillo Loeza, D. E., López, M. & Becerril García, J. La lucha socioambiental de proyectos alternativos. El caso del cerdo pelón en Yucatán. *Ecología Política. Cuadernos de debate internacional* **61,** 74–79 (2021).

La Sánchez Domónguez-Guilarte, M. d. C. El patrimonio wixarika: historia, memoria y lucha política en el contexto del multiculturalismo. *Revista Euroamericana de Antropología* **9,** 105 (2020).

La Torre-Cuadros, María de los Ángeles. Nota Científica: Hacia un Enfoque Biocultural en los Programas de Conservación de la Naturaleza. *Etnobiología* **11,** 53–57 (2013).

Lewis, L. *et al.* Cultivando un jardín de nombres en los bosques en miniatura del cabo de hornos. Extensión de la conservación biocultural y la ética a seres vivos poco percibidos. *Magallania* **46,** 103–123 (2018).

López, M. La decolonialidad como alternativa para la conservación de la biodiversidad. El caso de la meliponicultura en la Península de Yucatán. *Penìnsula* **16,** 29–53 (2021).

López, M., Gamiño, M. & Pinkus, M. A. La meliponicultura en la Reserva de la Biósfera de Los Petenes en la península de Yucatán, México. Una iniciativa decolonial. *Ecología Política. Cuadernos de debate internacional* **60,** 84–88 (2020).

López-García, A. *et al.* Conocimiento tradicional de hongos de importancia biocultural en siete comunidades de la región chinanteca del estado de Oaxaca, México. *Scientia Fungorum* **50,** 1-13 (2020).

Lucio, C. Mezcales y diversidad biocultural en los alrededores del Volcán de Colima. El caso de los productores tradicionales de Zapotitlán de Vadillo. *Entre Diversidades: Revista de Ciencias Sociales y Humanidades* **5,** 13–43 (2015).

Luque Agraz, D. & Doode Matsumoto, O. S. Los comcáac (seri): hacia una diversidad biocultural del Golfo de California y estado de Sonora, México. *Hermosillo, Son.* **17,** 273–301 (2009).

Luque, D. *et al.* Pueblos indígenas de Sonora: el agua, ¿es de todos? *región y sociedad* **3** (2016).

Luque, D. *et al.* Política ambiental y territorios indígenas de Sonora. *Estudios Sociales. Revista de Alimentación Contemporánea y Desarrollo Regional* **2,** 257–280 (2012).

Madrigal Calle, B. E., Escalona Maurice, M. & Vivar Miranda, R. Del meta-paisaje en el paisaje sagrado y la conservación de los lugares naturales sagrados. *Sociedad y Ambiente* **1,** 1–25 (2016).

Maldonado Ibarra, O. A., Chávez Dagostino, R. M. & Bravo Olivas, M. L. Áreas naturales protegidas y participación social en América Latina: problemas y estrategias para lograr la integración comunitaria. *región y sociedad* **32,** 1-24 (2020).

Malebrán, J. & Rozzi, R. Análisis de los cursos de filosofía ambiental de campo en el parque etnobotánico omora, reserva de la biosfera Cabo de Hornos, Chile. *Magallania* **46,** 207–225 (2018).

Mancera-Valencia, F. J. Los paisajes culturales del noroeste de Chihuahua: la región Casas Grandes-Paquimé. *Chihuahua Hoy* **17,** 203–258 (2019).

Mancera-Valencia, F. J., Ávila Reyes, A. A. & Amador Guzmán, P. M. Educación y patrimonio biocultural. Construcción de una experiencia en la educación indígena de la sierra Tarahumara. *IE Revista de investigación educativa de la REDIECH* **9,** 119–132 (2018).

Marchant Santiago, C., Fuentes Acuña, N., Kaulen Luks, S. & Tomás Ibarra, J. Saberes locales en huertas de montaña del sur de los Andes: un refugio de memoria biocultural mapuche pewenche. *Pirineos* **175,** 1–16 (2020).

Mariaca, K. Sentires y pensares epistemológicos sobre diversidad biocultural de montaña y desarrollo integral para Vivir Bien en Bolivia. *Revista Ciencia, Technología e Innovación* **17,** 11–30 (2019).

Martínez Coria, R. & Haro Encinas, J. A. Derechos territoriales y pueblos indígenas en méxico:. una lucha por la soberanía y la nación. *Revista Pueblos y fronteras digital* **10,** 228–256 (2015).

Martínez González, P. & Corgos López-Prado, A. La pesca artesanal en Jalisco. Conflictos en torno a la conservación biocultural y la reproducción del capital. El caso de Careyitos. *Sociedad y Ambiente* **2,** 23–38 (2014).

Mastretta-Yanes, A. *et al.* Un programa para méxico de conservación y uso de la diversidad genética de las plantas domesticadas y sus parientes silvestres. *Revista Fitotecnia Mexicana* **42,** 321–334 (2020).

Medici, A. Metabolismo social con la naturaleza, pluralismo jurídico y derechos emergentes. *ABYA-YALA: Revists sobre acesso á justiça e direitos nas Américas* **2,** 101–116 (2018).

Medina, Y., Massardo, F. & Rozzi, R. Educación, ecoturismo y conservación biocultural en los bosques en miniatura del Cabo de Hornos. *Magallania* **48,** 183–211 (2020).

Mejía Martine, C. Efectos de los megaproyectos hidroeléctricos sobre la gastronomía indígena, ejemplo del municipio de San Felipe Usila, Oaxaca, México. *Iberoamérica Social: Revista-red de estudios sociales* **8,** 84–104 (2020).

Mesa, M. R. P. Concepciones de biodiversidad desde la perspectiva de la diversidad cultural. Tensiones y horizontes en la educación. *Escritos sobre la Biología y su enseñanza* **6,** 43–59 (2013).

Mesa, M. R. P. Miradas De La Biodiversidad Y La Diversidad Cultural. Una Reflexión A Propósito De La Enseñanza De Las Ciencias. *Tecné, Episteme y Didaxis: TED* **Extra, november** (2014).

Millaleo Hernández, S. Guarda de la Naturaleza: Conocimientos Ecológicos Tradicionales de los Pueblos Indígenas y Estrategias de Protección. *Cadernos de dereito actual* **13,** 2020–2230 (2020).

Montano, M. E., Sanabria-Diago, O. L., Manzano, R. & Quilindo, O. Ruta biocultural de conservación de las semillas nativas y criollas en el territorio indígena de Puracé, Cauca. *(Revista U.D.C.A Actualidad & Divulgación Científica* **24,** 1–8 (2021).

Montoya, A. *et al.* Los hongos conocidos por la comunidad Yuhmu de Ixtenco, Tlaxcala, México. *Scientia Fungorum* **49,** 1-15 (2019).

Montoya, D. & Toledo, V. M. Historia de la caficultura en Chiapas (1880-2010). Apuntes de una evolución social y ambiental. *Sociedad y Ambiente* **23,** 1–25 (2020).

Moreno-Calles, A. I., Toledo, V. M. & Casas, A. Los sistemas agroforestales tradicionales de México: Una aproximación biocultural. *Botanical Sciences* **91,** 375–398 (2013).

Munguía-Vázquez, Á., Cárdenas-Camargo, I. & Rangel-Villafranco, M. Uso y conocimiento de cactáceas en la comunidad otomí de El Alberto, en Ixmiquilpan (Hidalgo, México). *Ambiente y Desarrollo* **22** (2018).

Neira Brito, F. Representaciones de la naturaleza en la Amazonía ecuatoriana: ¿subsistencia local o conservación global? *Íconos* **10.2,** 57–65 (2006).

Nemogá, G. R. Diversidad biocultural. Innovando en investigación para la conservación. *Acta Biológica Colombiana* **21,** 311–319 (2016).

Núñez López, R. A. & Hekking, E. Los saberes de la lengua. *Tlahuizcalli* **7,** 41–48 (2021).

Nuñez-García, R. M., Fuente Carrasco, Fuente Carrasco, Mario Enrique & Venegas-Barrera, C. S. La avifauna en la memoria biocultural de la juventud indígena de la Sierra Juárez de Oaxaca, México. *Universidad y ciencia* **28,** 201–216 (2012).

Ojeda, J. *et al.* Interacciones bioculturales del pueblo yagán con las macroalgas y moluscos: Una aproximación desde la filosofía ambiental de campo. Una aproximación desde la filosofía ambiental de campo. *Magallania* **46,** 155–181 (2018).

Olvera, D. R. C. Guardianes Del Mar y Del Viento. El Conflicto Socioambiental Del Pueblo Ikoot En El Istmo de Tehuantepec. *Ecología Política* **60,** 68–72 (2020).

Olvera, M. M. C. & Maldonado, A. L. C. Oasis Sudcalifornianos: Paisajes bioculturales con elevada capacidad adaptativa a la aridez y potencial para la construcción de la sustentabilidad local. *Fronteiras* **6,** 217–239 (2017).

Pacheco Calderón, D. Conocimiento tradicional campesino, una posibilidad de visualizar lo rural a propósito de la enseñanza de la biología en contexto. *Biografia* **12,** 143–150 (2019).

Padilla-Mejìa, A. & Ramírez-Calvo, D. Las aves dentro de la memoria biocultural de la población del Cantón de Paraíso, Cartago, Costa Rica. *Zeledonia* **23,** 29–43 (2019).

Palacios Mena, D. & Moreno Rodallega, M. Medidas de mitigación y corrección que utiliza el Estado para subsanar los daños producidos por los entables mineros en el barrio El Oasis del municipio de Certegui, departamento de Chocó. *Dixi* **21,** 1–28 (2020).

Paño Yáñez, P. Escenarios de las economías de comunidades amazónicas shuar del Ecuador en tiempos de globalización. *Boletín Americanista* **70,** 229–249 (2020).

Patrick-Encina, G. & Bastida Muñoz, M. C. El resguardo colectivo del patrimonio bio-cultural como garantía de la resiliencia de los sistemas socio-ecológicos de los pueblos en el estado de México. *Ra Ximhai* **6,** 373–378 (2010).

Pérez Mesa, M. R. Concepciones de biodiversidad y prácticas de cuidado de la vida desde una perspectiva cultural. *Tecné. Episteme. Didaxis: TED* **45,** 17–34 (2019).

Pérez, D. & Matiz-Guerra, L. C. Uso de las plantas por comunidades campesinas en la ruralidad de Bogotá D.C., Colombia. *Caldasia* **39,** 68–78 (2017).

Pérez, D., Mora, R. & López Carrascal, C. Conservación de la diversidad de yuca en los sistemas tradicionales de cultivo de la Amazonía. *Acta Biológica Colombiana* **24,** 202–212 (2019).

Ponce Valadez, M. Creando conciencia ecológica en el estudiante del bachillerato a distancia B@UNAM. *Revista Mexicana de Bachillerato a Distancia* **9,** 139–146 (2017).

Puentes, J. P., Arenas, P. M. & Hurrell, J. A. Lamiaceae medicinales y aromáticas comercializadas en el Área Metropolitana de Buenos Aires, Argentina. *Bonplandia* **29,** 5–20 (2020).

Quiñones Diaz, X. E., Muñoz Concha, D. & Aguilera Fernández, N. F. Comunidades campesinas, patrimonio agrario y mercados en los cultivos del ají y la quinoa. *Revista Pensamiento y Acción Interdisciplinaria* **7,** 112–128 (2021).

Ramírez García, R., Escudero Valdemoros, B., Escudero Fonseca, F., Pérez Moracia, F. & Fernández Rico, A. I. Nalda (La Rioja) apuesta por otra forma de turismo con la “comunidad cuidadora” como eje vertebrador. *revista PH* **27,** 222–231 (2019).

Ramírez Hernández, N. E. & Leguizamon Arias, W. Y. La naturaleza como víctima en la era del posacuerdo colombiano. *El Ágora USB* **20,** 260–274 (2020).

Ramos Muñoz, D. E. Sustentabilidad y patrimonio biocultural en la Reserva de la Biosfera del Ocote. *Trace* **74,** 9–37 (2018).

Ramos Roca, E. & Corona-M, E. La importancia de diversas, complementarias y comparativas miradas en la investigación sobre las interacciones entre los humanos y la fauna en América Latina. *Antípoda. Revista de Antropología y Arqueología* **28,** 13–29 (2017).

Rodríguez Caguana, A. & Morales Naranjo, V. Los derechos de la naturaleza en diálogo intercultural: una mirada a la jurisprudencia sobre los páramos andinos y los glaciares indios. *Deusto Journal of Human Rights* **6,** 99–123 (2020).

Rodríguez Ramírez, M. d. C., Aldasoro Maya, E. M., Zamora Lomelí, C. B. & Velasco Orozco, J. J. Conocimiento y percepción de la avifauna en niños de dos comunidades en la selva Lacandona, Chiapas, México: hacia una conservación biocultural. Hacia una conservación biocultural. *Nova Scientia* **9,** 660–716 (2017).

Román Suárez, H. R. Grupos y estrategias para la defensa biocultural del territorio en Felipe Carrillo Puerto, Quintana Roo. *Alteridades* **31,** 93–106 (2021).

Romero Ugalde, M. Caldos para el Xont’e. La territorialidad simbólica como reto legislativo en Guanajuato. *Acta Universitaria* **26,** 109–118 (2017).

Rossetti, T. Dinámicas Eco protectora: Sanación y Apache en Nandayuri como sistema de buen vivir. *Revista Nicaragüense de Antropología* **3,** 47–55 (2019).

Rozzi, R. Hacia una ética biocultural planetaria interregional: filosofía ambiental de campo y estudios socio-ecológicos a largo plazo desde el Cabo de Hornos, Chile. *Socializar conocimientos* **2,** 45-35 (2014).

Rozzi, R. Bioética Global y Ética Biocultural. *Cuadernos de bioetica* **27,** 339–355 (2016).

Rozzi, R. La filosofía ambiental de campo y la ecorregión subantártica de magallanes como un laboratorio natural en el antropoceno. *Magallania* **46,** 7–15 (2018).

Rozzi, R. *et al.* Filosofía ambiental de campo y conservación biocultural en el Parque Etnobotánico Omora. Aproximaciones metodológicas para ampliar los modos de integrar el componente social ("S") en Sitios de Estudios Socio-Ecológicos a Largo Plazo (SESELP). *Revista chilena de historia natural* **83,** 27–68 (2010).

Rozzi, R. *et al.* Un centinela para el monitoreo del cambio climático y su impacto sobre la biodiversidad en la cumbre austral de América: La nueva red de estudios a largo Plazo Cabo de Hornos. *Anales del Instituto de la Patagonia* **48,** 45–81 (2020).

Rozzi, R. *et al.* Árboles Nativos y Exóticos en las Plazas de Magallanes. Native and Exotic tree Species in the Squares of the Magellan Region. *Anales Instituto Patagonia* **31,** 27–42 (2003).

Rozzi, R. *et al.* Filosofía ambiental de campo: ecología y ética en las redes LTER-Chile e ILTER. *Bosque (Valdivia)* **35,** 439–447 (2014).

Rozzi, R. & Schüttler, E. Primera década de investigación y educación en la Reserva de la Biosfera Cabo de Hornos: El enfoque biocultural del Parque Etnobotánico Omora. El enfoque biocultural del Parque Etnobotánico Omora. *Anales Instituto Patagonia (Chile)* **43,** 19–43 (2015).

Ruiz-Barajas, C. A. Patrimonio, paisaje y resiliencia. Un encuentro en lo colectivo. *Revista Digital de Ciencias Sociales* **5,** 321–334 (2018).

Salas, M. A. & Tillmann, T. El poder transformador de los saberes en paisajes de terrazas. *Vegueta. Anuario de la Facultad de Geografía e Historia* **21,** 267–301 (2021).

Sánchez-Zárate, P. U. Diseño de servicios: una estrategia para el etnoturismo. *Gestión y Ambiente* **19,** 289–301 (2016).

Santos Tanús, A., Aldasoro Maya, E. M., Rojas Serrano, C. & Morales, H. Especies Alimenticias de Recolección y Cultura Culinaria: Patrimonio Biocultural de la comunidad popoloca Todos Santos Almolonga, Puebla, México. *Nova Scientia* **11,** 296–342 (2019).

Scarpa, G. F. & Rosso, C. N. Etnobotánica histórica de grupos Criollos de Argentina IV: Identificación taxonómica de las plantas y análisis de datos medicinales del Chaco Húmedo provenientes de la Encuesta Nacional de Folklore de 1921. *Bonplandia* **28,** 5–42 (2018).

Socies Fiol, A. & Cuéllar Padilla, M. ¿Quién mantiene la memoria biocultural y la agrobiodiversidad en la isla de Mallorca? Algunos aprendizajes desde las variedades locales de tomate. *Revista de Dialectología y Tradiciones Populares* **72,** 477-50 (2017).

Tetreault, D. V. & Lucio López, C. F. Jalisco: pueblos indígenas y regiones de alto valor biológico. *Estudios sobre Estado y Sociedad* **18,** 165–199 (2011).

Thomé-Ortiz, H. & García-Soto, E. A. La dimensión recreativa de los hongos comestibles silvestres de Senguio Michoacán, México, y sus escenarios de desarrollo local. *Agro Productividad* **12,** 45–50 (2019).

Toledo Manzur, V. M. El paradigma biocultural: crisis ecológica, modernidad y culturas tradicionales. *Sociedad y Ambiente* **1,** 50–60 (2013).

Torrealba, I. & Carbonell, F. La Conservación Integral Alternativa desde el Sur: una visión diferente de la conservación. *Polis (Santiago)* **7,** 339–363 (2008).

Torres Villa, R. & Barragán López, E. Hábitat de la cultura ranchera en la sierra de Jalisco y Michoacán, México. Potencial para el aprovechamiento de un turismo biocultural. *International journal of scientific management and tourism* **2,** 281–301 (2015).

Torrescano Valle, N. *et al.* Percepción comunitaria de las áreas protegidas, a más de 30 años de su creación en Ecuador. *Trace* **74,** 60–91 (2018).

Vásquez González, A. Y., Chávez Mejía, C., Herrera Tapia, F. & Meléndez, F. C. La fiesta xita: patrimonio biocultural mazahua de San Pedro el Alto, México. *Culturales* **4,** 199–228 (2016).

Vásquez González, A. Y., Chávez Mejía, M. C., Herrera Tapia, F. & Carreño Meléndez, F. La milpa mazahua: baluarte de conocimientos y creencias. *Iberofórum. Revista de Ciencias Sociales de la Universidad Iberoamericana.* **11,** 142–167 (2016).

Vásquez González, A. Y., Chávez Mejía, C., Herrera Tapia, F. & Carreño Meléndez, F. Milpa y seguridad alimentaria: El caso de San Pedro El Alto, México. *Revista de Ciencias Sociales (Ve)* **24** (2018).

Vásquez-García, A., Sangerman-Jarquín, D. M. & Schwentesius Rindermann, R. Caracterización de especies de abejas nativas y su relación biocultural en la Mixteca oaxaqueña. *Revista Mexicana de Ciencias Agrícolas* **12,** 101–113 (2021).

Vicente Giménez, T. De la justicia climática a la justicia ecológica: los derechos de la naturaleza. *Revista Catalana de Dret Ambiental* **11,** 1–42 (2020).

Victoria Ojeda, J. El arribo de los españoles a la Peninsula de Yucatán y el inicio del cambio en el paisaje biocultural de la región, siglo XVI. *Boletín Americanista* **69,** 175–195 (2019).

Villalba, M. S. & Lambaré, D. A. Las manzanas en Ocumazo (Jujuy, Argentina) como parte de la diversidad de cultivos tradicionales: usos y percepciones. *Boletín De La Sociedad Argentina De Botánica* **54,** 431–449 (2019).

Xochit, J. A. Análisis y perspectivas para gestionar el turismo biocultural: una opción para conservar el ecosistema forestal de Temascaltepec. *Madera y Bosques* **24,** 1–14 (2018).

Zárate Ángel, D. A., Cantú Chapa, R., Silva García, J. T. & Hernández Suárez, Y. Movimientos sociales, conservación del paisaje biocultural y ecoturismo solidario en el corredor Mazunte-Escobilla, Oaxaca, México. *UVserva* **9,** 158–172 (2020).

Zent, E., Zent, S. & Marius, L. Autodemarcando la Tierra. Explorando ideas, árboles y caminos Hotï. *Boletín Antropológico* **21,** 313–338 (2003).

Appendix S2

Correlations in the data

Table S1. Results of the correlation test of the quantitative variables with the ordination space of the DCA of the lens assignments. P-values are based on a permutation test with 9999 permutations and a significance level of 0.05. R^2 is the squared correlation coefficient

Variable	DCA1	DCA2	R^2	P-value	Significance
Gender	-0.03	-1	0.05	0.022	*
Transdisciplinarity	-0.81	0.58	0.04	0.072	
Instrumental value	-0.96	-0.27	0.09	0.001	**
Relational value	0.94	-0.34	0.03	0.115	
Intrinsic value	0.71	0.7	0.15	<0.001	***
Conceptual	0.97	0.22	0.07	0.009	**
Discussion	1	-0.07	0.06	0.018	*
Empirical	-0.96	-0.28	0.14	<0.001	***
Review	0.66	-0.75	0.01	0.645	
Transformation	0.85	-0.53	0.03	0.142	
Bottom-up governance	-1	-0.09	0.05	0.024	*
Top-down governance	0.99	0.13	0.02	0.229	
Action	0.77	0.64	0.03	0.126	
Cultural	0.59	-0.81	0.04	0.057	
Traditional knowledge	-0.54	-0.84	0.06	0.015	*

Appraising biocultural approaches to sustainability in the scientific literature in Spanish

Isabel Díaz-Reviriego e-mail: diaz@leuphana.de

Isabel Díaz Reviriego is a postdoctoral researcher at the Social-Ecological Systems Institute, Leuphana University of Lüneburg. Her research interests include biocultural diversity, equity and inclusion in sustainability research and science-policy interfaces, and feminist political ecology.

Social-Ecological Systems Institute (SESI), Faculty of Sustainability, Leuphana University of Lüneburg, Lüneburg, Germany

Jan Hanspach e-mail: hanspach@leuphana.de

Jan Hanspach is a Junior Research Group Leader at the Social-Ecological Systems Institute, Leuphana University of Lüneburg. His research interests include biocultural diversity, biodiversity conservation, and scenario planning in farming landscapes.

Social-Ecological Systems Institute (SESI), Faculty of Sustainability, Leuphana University of Lüneburg, Lüneburg, Germany

Mario Torralba e-mail: m.torralbaviorreta@vu.nl

Mario Torralba is an assistant professor at the Environmental Geography Group of the IVM Institute for Environmental Studies, VU Amsterdam. His research interests include sustainable landscape management, biodiversity conservation, and participatory research methods.

Environmental Geography Group, IVM Institute for Environmental Studies, VU University Amsterdam, Amsterdam, The Netherlands

Stefan Ortiz-Przychodzka e-mail: ortiz@leuphana.de

Stefan Ortiz-Przychodzka is a Research Associate and PhD candidate at the Social-Ecological Systems Institute, Leuphana University of Lüneburg. His research interests include biocultural diversity, political ecology, relational values, and pluralist economies in rural landscapes.

Social-Ecological Systems Institute (SESI), Faculty of Sustainability, Leuphana University of Lüneburg, Lüneburg, Germany

Camila Benavides Frias e-mail: camila.benavides_frias@leuphana.de

Camila Benavides-Frias is a research associate and doctoral candidate at the Social- Ecological Systems Institute, Leuphana University of Lüneburg. Her research interests include biodiversity conservation, food systems, and environmental education.

Social-Ecological Systems Institute (SESI), Faculty of Sustainability, Leuphana University of Lüneburg, Lüneburg, Germany

Leonie Burke e-mail: leonie.burke@students.uni-freiburg.de

Leonie Burke is a student assistant at the Social-Ecological Systems Institute, Leuphana University of Lüneburg and a Master's student of Geography of Global Change at Albert-Ludwigs-Universität Freiburg. Her research interests include biocultural diversity, political ecology, and environmental justice.

Social-Ecological Systems Institute (SESI), Faculty of Sustainability, Leuphana University of Lüneburg, Lüneburg, Germany

María García-Martín e-mail: maria.garcia-martin@wsl.ch

María García-Martín is a postdoctoral researcher at the Land Change Science unit of the Swiss Federal Institute for Forest, Snow and Landscape Research, WSL. Her research interests include integrated landscape management approaches and the study of the personal interaction with places for cultivating landscape stewardship.

Land Change Science Research Unit, Swiss Federal Research Institute WSL, Birmensdorf, Switzerland

Elisa Oteros-Rozas e-mail: elisa.oterosrozas@gmail.com

Elisa Oteros-Rozas is a postdoctoral researcher and professor at the Agronomy Department of the Higher Technical School of Agronomic Engineering of the University of Seville, and member of FRACTAL Collective. er research interests include agroecology, pastoralism,
local/traditional ecological knowledge, biodiversity conservation, scenario planning, and gender studies.

University of Seville, Spain. FRACTAL Collective, Spain

Corresponding Author:
Isabel Díaz Reviriego
diaz@leuphana.de
Tel: +49 160 5412439

Word Count: 10.833 words including abstract, *resúmen* and references

Acknowledgements

We would like to appreciate the insights and direction provided by Berta Martín-López at the initial conception of the manuscript. We are also grateful to Kristina Raab for editing the English, Ana Varela for graphic support and Mercedes Dilara for data management support.

Conflict of interest

Authors declare no conflict of interest

Funding

This work was supported by the German Ministry for Education and Research (BMBF) within the framework of the Strategy „Research for Sustainability" (FONA; www.fona.de/en) as part of its Social-Ecological Research funding priority (Grant No. 01UU1903)
